# Growth evolution and phase transition from chalcocite to digenite in nanocrystalline copper sulfide: Morphological, optical and electrical properties

**DOI:** 10.3762/bjnano.5.166

**Published:** 2014-09-15

**Authors:** Priscilla Vasthi Quintana-Ramirez, Ma Concepción Arenas-Arrocena, José Santos-Cruz, Marina Vega-González, Omar Martínez-Alvarez, Víctor Manuel Castaño-Meneses, Laura Susana Acosta-Torres, Javier de la Fuente-Hernández

**Affiliations:** 1 Posgrado en Ciencia e Ingeniería de Materiales, Centro de Física Aplicada y Tecnología Avanzada, Universidad Nacional Autónoma de México, 76230, Querétaro, México; 2 Escuela Nacional de Estudios Superiores Unidad León, UNAM, Boulevard UNAM No. 2011 Predio el Saucillo y el Potrero, 36969, León Guanajuato, México; 3 Facultad de Química, Materiales Universidad Autónoma de Querétaro, 76010, Querétaro, México; 4 Centro de Geociencias, UNAM, 76230, Querétaro, México; 5 Departamento de Ingeniería en Energía, Universidad Politécnica de Guanajuato, 38483, Guanajuato, México; 6 Departamento de Ingeniería Molecular de Materiales, CFATA, UNAM, 76230, Querétaro, México

**Keywords:** abundant materials in the crust of Earth, electrical resistance, nanocrystals, nanodisks, non-toxic semiconductors, optical band gap, phase transition, photocurrent

## Abstract

Copper sulfide is a promising p-type inorganic semiconductor for optoelectronic devices such as solar cells, due its small band gap energy and its electrical properties. In this work nanocrystalline copper sulfide (Cu*_x_*S), with two stoichiometric ratios (*x* = 2, 1.8) was obtained by one-pot synthesis at 220, 230, 240 and 260 °C in an organic solvent and amorphous Cu*_x_*S was obtained in aqueous solution. Nanoparticle-like nucleation centers are formed at lower temperatures (220 °C), mixtures of morphologies (nanorods, nanodisks and nanoprisms) are seen at 230 and 240 °C, in which the nanodisks are predominant, while big hexagonal/prismatic crystals are obtained at 260 °C according to TEM results. A mixture of chalcocite and digenite phases was found at 230 and 240 °C, while a clear transition to a pure digenite phase was seen at 260 °C. The evolution of morphology and transition of phases is consistent to the electrical, optical, and morphological properties of the copper sulfide. In fact, digenite Cu_1.8_S is less resistive (346 Ω/sq) and has a lower energy band gap (1.6 eV) than chalcocite Cu_2_S (5.72 × 10^5^ Ω/sq, 1.87 eV). Low resistivity was also obtained in Cu*_x_*S synthesized in aqueous solution, despite its amorphous structure. All Cu*_x_*S products could be promising for optoelectronic applications.

## Introduction

Metallic chalcogenides based on cadmium, such as cadmium telluride, CdTe, or cadmium sulfide, CdS, have been widely investigated regarding their application in the optoelectronic field, mainly in photovoltaic devices due to the semiconducting, electronic and optical properties [1–5]. Cadmium is a toxic heavy metal, which limits its applications in the optoelectronic area. In fact, the current trend is to develop environment-friendly nanometric semiconductors with adequate optoelectronic properties for solar cells. It is well known that all properties (physical, chemical, magnetic) of nanometric materials differ from the bulk semiconductor due to the quantum effects [6]. Among the non-toxic nanomaterials with a small energy band gap that are promising for photovoltaic devices are: iron sulfide (FeS_2_), tungsten sulfide (WS_2_) and copper sulfide (Cu_2_S) [7]. The last is a terrestrially abundant and interesting semiconductor due to its stoichiometric variety usually depicted as Cu*_x_*S. Copper-rich sulfides (Cu_2_S), Cu*_x_*S with *x* = 0.03, 0.2, 0.25, and CuS are widely reported [8–27]. The stoichiometric ratio can be tailored by changing the concentration of copper or sulfide precursors, the reaction parameters and the kind of solvents. The following phases were obtained: djurleite (Cu_1.97_S), digenite (Cu_1.8_S) or analite (Cu_1.75_S) [8–29]. These crystalline phases are stable p-type compounds, which could be used as absorber materials in solar cells [30–32]. However, the exact identification of the crystalline structure is controversial due to the stock of 86 XRD patterns for Cu*_x_*S, some of which have reflections with narrowly spaced positions (see Table 1). This proximity makes it difficult to clearly assign diffraction patterns to certain crystalline phases.

**Table 1:** Crystalline phases of copper sulfide from copper-rich (Cu_2_S) to the lower concentration of copper (CuS) prepared in organic and aqueous media reported in the literature [8–27].

JCPDS	crystalline structure	morphology	position of reflections in [2θ, °] (respective crystallographic planes)	band gap energy *E*_g_ (eV)	solution/reference

84-0209	β-Cu_2_S	bulk crystals	37.5 (1 0 2); 45.5 (1 1 0); 48 (1 0 3); 54 (0 0 4); 54.5 (2 0 1)	1.22	organic/[8]
232-0961	ortho. α-Cu_2_S	films	27.5 (1 8 0); 33 (0 4 4); 47 (2 13 1); 51 (5 9 3); 57 (6 0 5)	2.48^a^	aqueous/[9]
02-1294	ortho. α-Cu_2_S	hexagonal nanodisks	≈38; ≈46; ≈48.5; ≈61		organic/[10]
84-1770	Cu_2_S	films	27.5 (1 1 1); 32.3 (2 0 0); 46 (2 2 0); 54.5 (3 1 1)		aqueous/[11]
00-0649	Cu_2_S	hexagonal nanodisks	≈37.5; ≈45.5; ≈48.5; ≈54.5		organic/[12]
26-1116	hex. β-Cu_2_S	14-facets polyhedra	37.5 (1 0 2); 45.5 (1 1 0); 48 (1 0 3); 54 (1 1 2)	1.35–1.56^b^	organic/[13]
26-1116	hex. β-Cu_2_S	hexagonal nanocrystals	34.5 (1 0 1); 44 (1 0 2); 54 (1 1 0); 57 (1 0 3); 64.5 (0 0 4)		organic/[14]
26-1116	hex. β-Cu_2_S	hexagonal nanodisks	37.5 (1 0 2); 45.5 (1 1 0); 48 (1 0 3); 54 (1 1 2)	1.36–1.53^b^	organic/[15]
26-1116	hex. β-Cu_2_S	hexagonal nanodisks	37.5 (1 0 2); 45.5 (1 1 0); 48 (1 0 3); 54.5 (0 0 4); 56 (2 0 1)		water–organic/[16]
26-1116	hex. β-Cu_2_S	nanorods	26.5 (0 0 2); 30 (1 0 1); 37.5 (1 0 2); 46 (1 1 0); 48.5 (1 0 3); 59 (2 0 0)	2.48^a^	water–organic/[17]
29-0578	tet. Cu_1.96_S	films	27.5 (1 0 2); 32.5 (1 0 3); 39 (1 0 4); 48.5 (2 0 2)	1.4	aqueous/[9]
04-0842	digenite Cu_1.8_S	spherical nanoparticles	≈28; ≈32.5; ≈46.5		organic/[12]
47-1748	digenite Cu_1.8_S	irregular nanoparticles	≈28; ≈32; ≈46.5		aqueous/[18]
47-1748	digenite Cu_1.8_S	irregular nanoparticles	≈28; ≈32; ≈46.5; ≈55		water–organic/[19]
24-0061	digenite Cu_1.8_S	films	28 (1 1 1); 32 (2 0 0); 46 (2 2 0)	1.55	aqueous/[20]
23-0960	cub. digenite Cu_1.76_S	films	28 (6 6 2); 32 (8 6 0); 47 (10 10 0); 55 (15 7 1)	2.11^a^	aqueous/[9]
00-0833	CuS	spherical nanoparticles	≈29.5; ≈32.5; ≈48.5		organic/[12]
79-2321	CuS	irregular nanoparticles	29.5; 32; 48; 59		aqueous/[18]
01-1281	hex. CuS	clusters of nanoparticles	29 (1 0 2); 32 (1 0 3); 48 (1 1 0); 52.5 (1 0 4);		aqueous/[21]
03-0724	hex. CuS	nanoflowers	27.6 (1 0 1); 29.5 (1 0 2); 31.6 (1 0 3); 47.6 (1 1 0); 52.5 (1 0 8); 59 (1 1 6)		aqueous/[22]
85-0620	CuS	films based on nanorods	44 (1 0 6); 45 (0 0 8); 51 (1 0 8); 54 (1 1 6); 65 (2 1 6); 75 (2 0 8)		aqueous/[23]
06-0464	hex. CuS	films	29 (1 0 2); 31.5 (1 0 3); 32.5 (0 0 6); 48 (1 1 0); 52.5 (1 0 8)	1.72^a^	aqueous/[9]
06-0464	hex. CuS	films	29 (1 0 2); 31.5 (1 0 3); 32.5 (0 0 6); 48 (1 1 0)	1.55	aqueous/[20]
06-0464	hex. CuS	films	32 (1 0 3); 39.5 (1 0 5); 43.5 (1 0 6); 48 (1 1 0); 53 (1 0 8); 59.5 (1 1 6); 74.5 (2 0 8)	2.8	organic/[24]
06-0464	CuS	films based on polycrystals	28 (1 0 1); 29 (1 0 2); 32 (1 0 3) 34 (0 0 6); 48 (1 1 0); 59 (1 1 6); 59.2 (1 0 6) 52 (1 0 8)	2^a^; 2.58^b^	aqueous/[25]
06-0464	hex. CuS	nanoflowers	27.6 (1 0 1); 29.5 (1 0 2); 31.6 (1 0 3); 48 (1 1 0); 52.5 (1 0 8); 59 (1 1 6)		ethanol/[26]
06-0464	hex. CuS	irregular nanoparticles	28 (1 0 1); 29 (1 0 2); 32 (1 0 3); 48 (1 1 0); 52 (1 0 8); 59 (1 1 6)		water–oil/[27]

^a^Direct band gap; ^b^indirect band gap.

On the other hand, the control of size, shape, distribution and stoichiometry of Cu*_x_*S is an essential challenge nowadays, because these parameters are dependent on several factors [12–13,15,18,21]. For example, the reaction temperature modified the shape, size and optical properties of monodisperse Cu_2_S obtained from a simple one-pot route [15]. In fact, there exists wide research about the synthesis of copper sulfide nanostructures obtaining different Cu/S ratios [9,11,16,20,23–26]. However, the lack of knowledge about the growth evolution and the phase transitions of copper sulfide is the motivation of this work.

In this work, the growth evolution and the phase transition of copper sulfide in the temperature range from 220 to 260 °C in an organic solvent is reported. The full electrical, morphological and optical properties of these crystalline samples synthesized in the organic solvent were compared with the amorphous Cu*_x_*S obtained from aqueous solution.

## Results and Discussion

### Structural properties from X-ray diffraction

**Figure 1:**
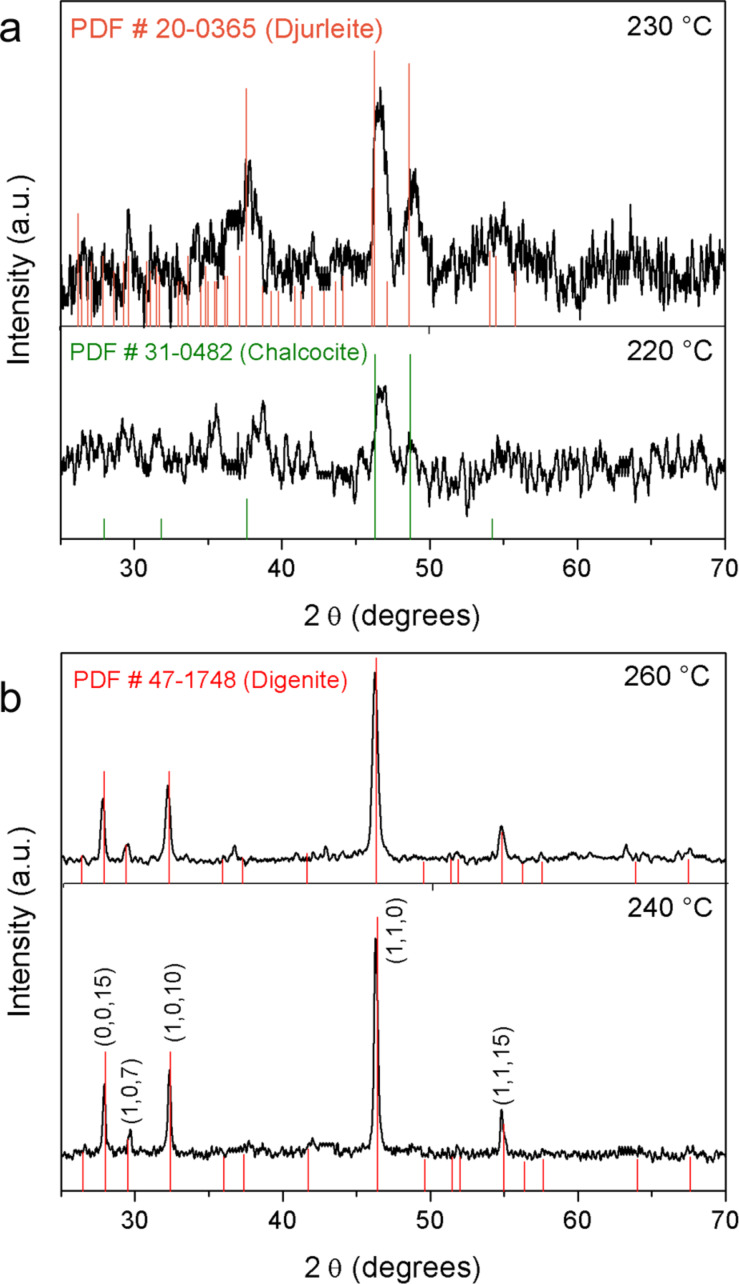
X-ray patterns of copper sulfide synthesized in organic solution at a) 230 and 220 °C, the chalcocite phase is obtained and at b) 240 and 260 °C, the predominant phase is the digenite.

The structural properties of the copper sulfide samples (Cu*_x_*S) depend on the synthesis and the reaction temperature (Figure 1). A fully amorphous product is obtained from aqueous solution according to the X-ray diffraction pattern (Figure S1 in Supporting Information File 1). However, the crystallinity of organic products is dependent on the temperature reaction. At 220 °C, Cu*_x_*S presents three peaks with low intensity at 2θ = 38, 46.5 and 49° corresponding to the chalcocite structure (JCPDS 31-0482) (Figure 1a). Above a temperature of 230 °C, the Cu*_x_*S product is more crystalline. There are four peaks with broadening and better intensity at 2θ = 37.84, 46.5, 48.82, and 54.94°, which match both to the chalcocite (JCPDS 31-0482) phase and djurleite phase (JCPDS 20-0365). At 240 °C (Figure 1b), well defined peaks of the digenite phase (Cu_1.8_S, JCPDS 47-1748) appear at 28.26, 30.02, 32.66, 42.42, 46.62, 52.32, and 55.12 corresponding to the rhombohedral structure, which is consistent to the literature [19]. Small peaks of chalcocite can be seen, which are indicative of a mixture of phases. The X-ray pattern of Cu*_x_*S synthesized at 260 °C presents sharp peaks at 2θ = 27.84, 32.22, 32.66, 46.24, 55.12, and 67° of the digenite phase.

Three shapes of unit cells of Cu_2_S chalcocite phase can be presented: monoclinic (low chalcocite), hexagonal (high chalcocite), and cubic (cubic chalcocite) [30]. It is well known that the transformation from monoclinic (α,γ-Cu_2_S) to hexagonal (β-Cu_2_S) occurs at 103.5 °C and 101.8 °C for bulk and nanostructure chalcocite, respectively [33]. According to Machani et al. [34] the monoclinic phase changes to djurleite in ambient air and the real phase obtained is djurleite instead of chalcocite, even though, the chalcocite phase is usually reported [8,12–15]. In fact, the djurleite phase is obtained in ambient air [18], while chalcocite is obtained under argon atmosphere [14]. So, the products reported here obtained at 220 °C and 230 °C really are the chalcocite phase despite some peaks which match with djurleite. In fact, the Cu*_x_*S products maintained the crystalline phases after we stored them for one year at room temperature, which is indicative of a good stability of the Cu_2_S chalcocite and Cu_1.8_S digenite phases (results not shown here).

The grain size and stress of the crystalline copper sulfide samples from organic synthesis at 230–260 °C were obtained from the full widths at a half maximum (FWHM) of the diffraction peaks and the linear combination of the following equation [35]:


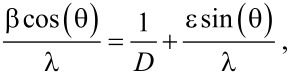


where β is the FWHM measured in radians, θ the Bragg angle of the peaks, λ the XRD wavelength, in our case in nanometers (λ = 0.154 nm), *D* is the effective crystallite size, and ε is the effective strain. A plot of β cos(θ)/λ versus sin(θ)/λ for all the samples gives the grain size and the strain, as shown in Figure S2 in Supporting Information File 1. The intercept is the inverse of the grain size and the slope is the strain, respectively. The grain size increases as the temperature increases (24.5 to 28.3 nm), the effective strain decreases in the samples shown that the least stress was at 260 °C (−8.26 × 10^−5^) and the highest was at 230 °C (−2.73 × 10^−3^).

### Morphology from TEM and HRTEM

TEM images revel that amorphous Cu*_x_*S from aqueous solution is constituted of nanometric particles with undefined shape that are agglomerated into clusters (See Figure S3 in Supporting Information File 1), which is in concordance with Cu*_x_*S obtained in similar aqueous systems [21].

**Figure 2:**
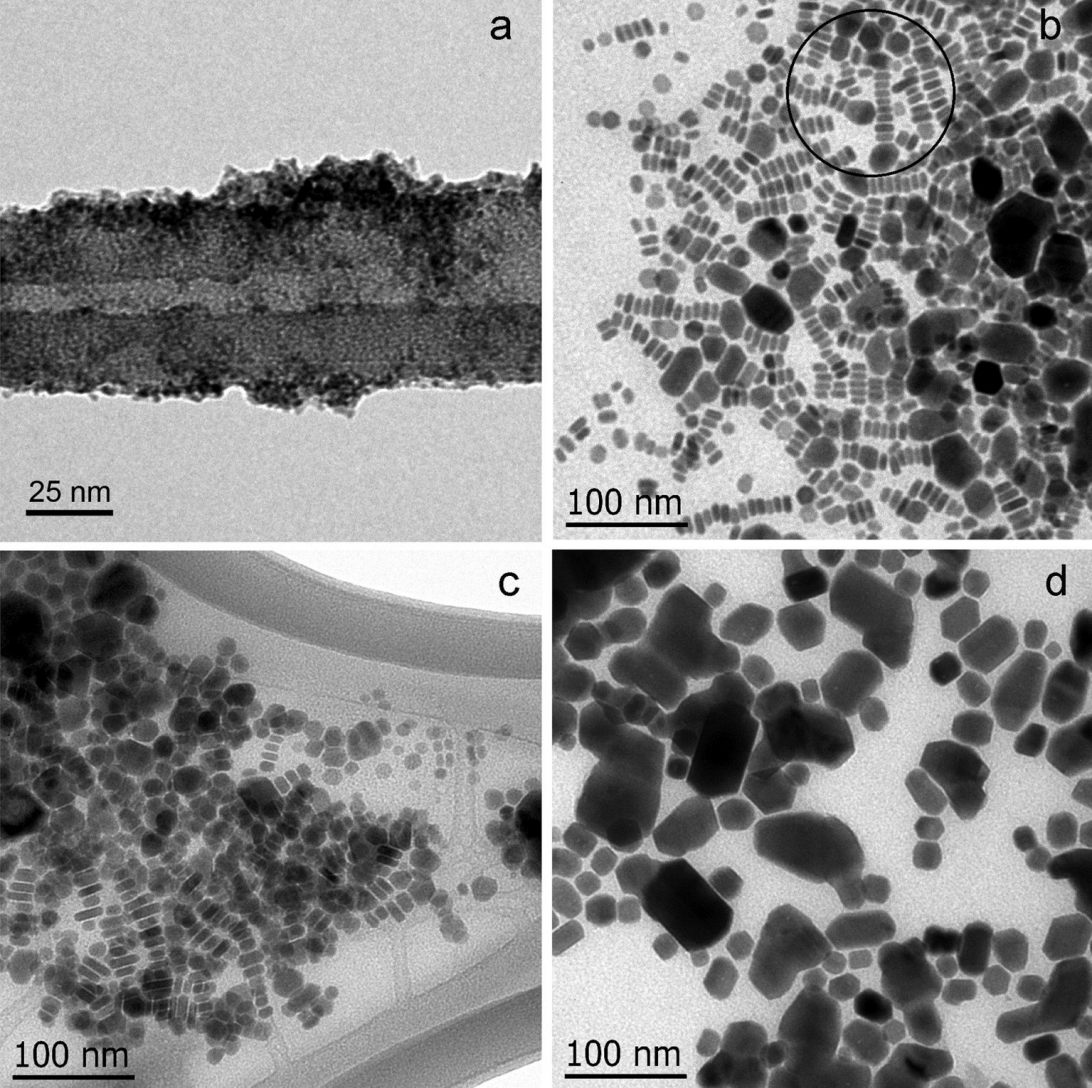
TEM images of copper sulfide synthesized in organic solution at a) 220, b) 230, c) 240 and d) 260 °C. The morphology of the Cu*_x_*S change from irregular nanoparticles to nanoprisms with increasing temperature. The encircled area shows an alignment of the nanorrods (b).

The morphology of Cu*_x_*S samples from organic solution depends on the reaction temperature, for example irregular particles below 10 nm can be observed for Cu*_x_*S obtained at 220 °C (Figure 2a). At 230 °C short chains of stacked nanorods with lengths (*l*) and width (*w*) of about 13.97 ± 2.7 × 5.86 ± 1.09 nm (from 260 particles), are seen in Figure 2b. Some hexagonal nanodisks of about 20–40 nm and prisms of about 50 nm are also observed. At 240 °C (Figure 2c) aligned nanorods are seen with similar dimensions (13.55 ± 1.86 × 5.91 ± 0.75 nm from 130 particles) to those seen at 230 °C. The size of the Cu*_x_*S crystals at the higher temperature is not significantly different. However, the amount of crystals with a prism geometry is increased. These two types of morphology are consistent to the mixture of phases that were shown in the X-ray results. Big crystals with different sizes (25–80 nm) are observed for the samples of Cu*_x_*S synthesized at 260 °C (Figure 2d) and a fewer nanorods of about 17.35 ± 3.70 × 6.59 ± 1.27 nm (from 30 particles) are also seen in Figure S4 in Supporting Information File 1. The average aspect ratios (*l*/*w*) of the Cu*_x_*S nanorods are about 2.38 (230 °C), 2.29 (240 °C), and 2.63 (260 °C) taken from the data of size distribution (Figure S5 in Supporting Information File 1). The change and evolution of the morphology is consistent to the transition of phase, from chalcocite to digenite.

**Figure 3:**
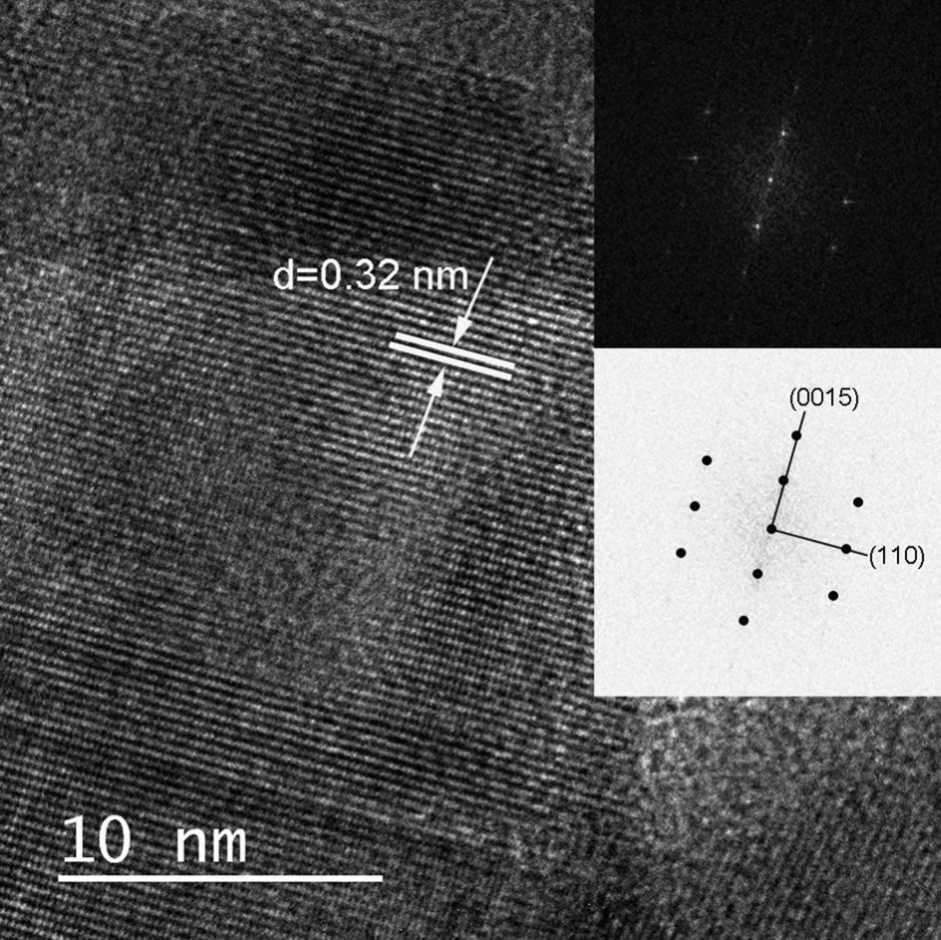
HRTEM image of copper sulfide obtained from synthesis in an organic solvent. The inset figures display the crystallographic planes (0015) and (110), respectively, of the digenite phase.

In order to verify the full transition of the digenite phase an HRTEM analysis of the crystals was made. The distance between the lines in the HRTEM image (Figure 3) is approximately 0.32 nm. This corresponds to the (0015) plane spacing of the digenite phase, which matches the peak of 46% of intensity in the XRD pattern shown in Figure 1b. The diffraction pattern of electrons obtained by the Fourier transformation (inset of Figure 3) shows an interplanar distance of about 0.197 nm, close to the value 0.19644 nm for the (110) spacing of the digenite phase (the peak for 100% intensity in the XRD pattern).

From TEM images, it can be observed that the phase transformation occurs from 220 to 260 °C and involves three stages: the nucleation, the shape evolution of the chalcocite crystals and the transition of the chalcocite to the digenite phase. Nanoparticles are formed in the first stage at 220 °C, which are the nuclei to the formation of a mixture of morphologies, i.e., nanodisks (25–40 nm) and irregularly shaped prisms (50–100 nm). The movement of the nanodisks results in the formation of the digenite phase through fusion of the nanodisks.

### Cu/S ratio from EDS

The EDS patterns shows two peaks at 0.9 and 8.0 keV attributed to Cu Kα and Cu Lα emission, while a third peak at 2.3 keV is due to the S Kα emission. Table 2 displays the average of Cu/S ratios calculated from the atomic percentage of each element from at least three measurements. The amorphous copper sulfide synthesized in an aqueous solution has a Cu/S ratio of 1.48 ± 0.03, close to the 1:1 ratio of CuS [18]. The organic Cu*_x_*S samples show the following Cu/S ratios: 1.58 ± 0.02 for the sample at 220 °C, 1.92 ± 0.05, and 1.83 ± 0.08 for crystalline chalcocite/digenite at 230 and 240 °C, respectively, and 1.69 ± 0.05 for the digenite phase (at 260 °C). These values are similar to the chalcocite Cu_2_S and digenite Cu_1.8_S phases, respectively.

**Table 2:** Summary of the morphological, optical, and electrical properties of Cu*_x_*S samples.

samples/temperature (°C)	crystalline phase	nanorod dimensions *l × w* (nm)	Cu/S ratio	maximum absorbance peak (nm)	direct *E*_g_ (eV)	resistance (Ω/sq)

A^a^/100	amorphous	—	1.48 ± 0.03	530	2.20	461.50
O^b^/220	chalcocite	—	1.58 ± 0.02	—	1.57	8.66 × 10^6^
O/230	chalcocite/ digenite	13.97 ± 2.7 × 5.86 ± 1.09	1.92 ± 0.05	440	1.87	5.72 × 10^5^
O/240	chalcocite/ digenite	13.55 ± 1.8 × 5.91 ± 0.75	1.83 ± 0.08	480	1.76	7.29 × 10^7^
O/260	digenite	17.35 ± 3.7 × 6.59 ± 1.27	1.69 ± 0.05	540	1.60	346.45

^a^Samples from aqueous solution; ^b^samples from organic solution.

### Optical properties

**Figure 4:**
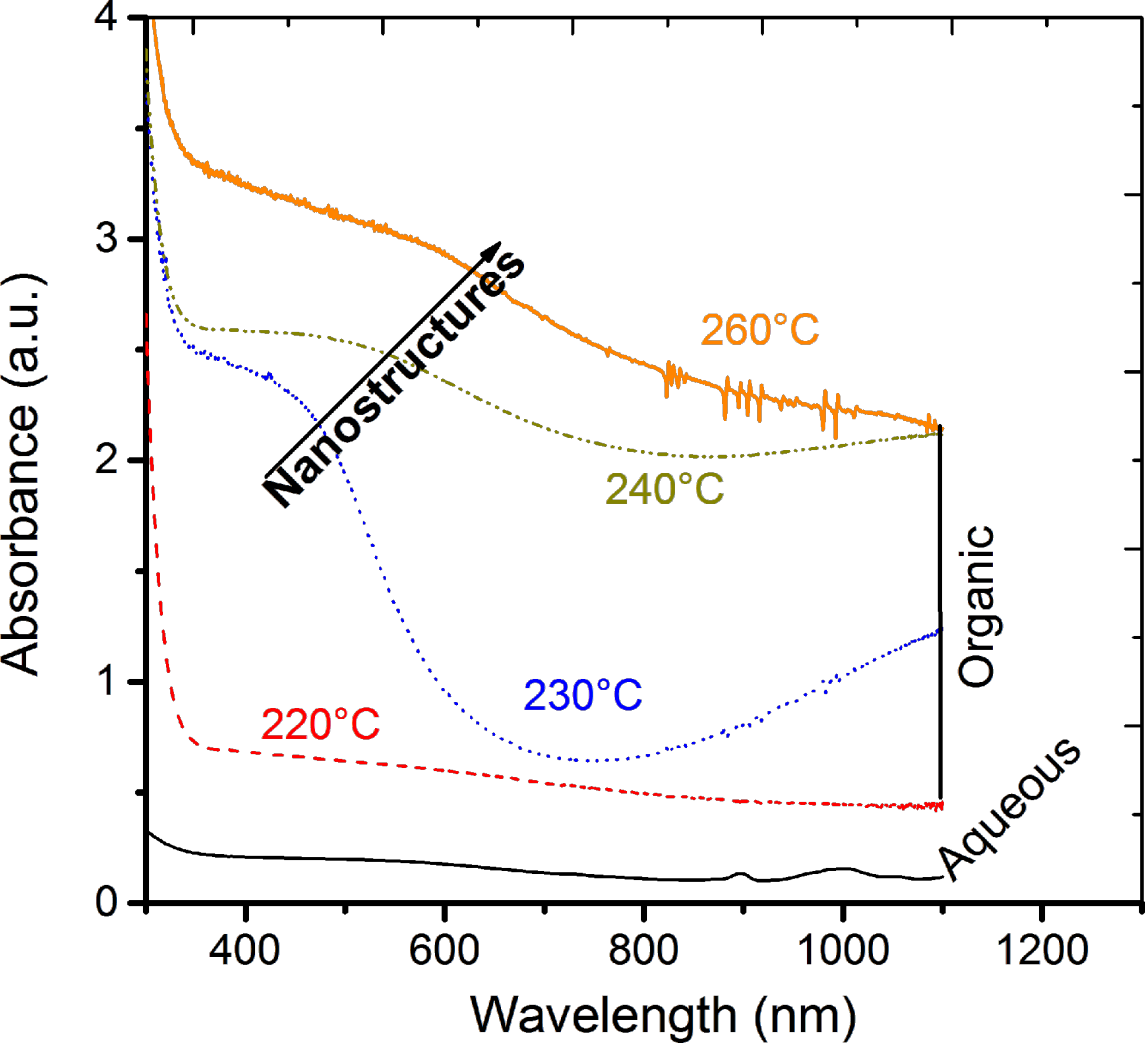
Absorbance of copper sulfide nanocrystals synthesized in an aqueous solution and in an organic solvent. A clear shift towards low energies is observed in the Cu_x_S samples synthesized in the range from 230 to 260 °C.

The optical absorbance spectra of the Cu*_x_*S are shown in Figure 4. Both, the amorphous sample from aqueous synthesis and the chalcocite Cu*_x_*S from organic synthesis at 220 °C, present a weak and broad absorption band at approximately 500 nm. However, crystalline Cu*_x_*S samples show a well-defined absorbance band between 490 to 600 nm. In fact, a red shift of about 40 to 60 nm is presented from the chalcocite (Cu_2_S) to the digenite phases (Cu_1.8_S), which is in agreement to the increment of crystal size. This phenomenon is related to the free charges due to the copper deficiency in the samples. For example, the maximum absorbance band has been reported at 450 nm for Cu_2_S, while it is observed at longer wavelength (950 nm) for CuS [36]. It is clear, that the deficiencies of copper generate a displacement or shift of the optical absorption, which is consistent to the transition of the phases.

**Figure 5:**
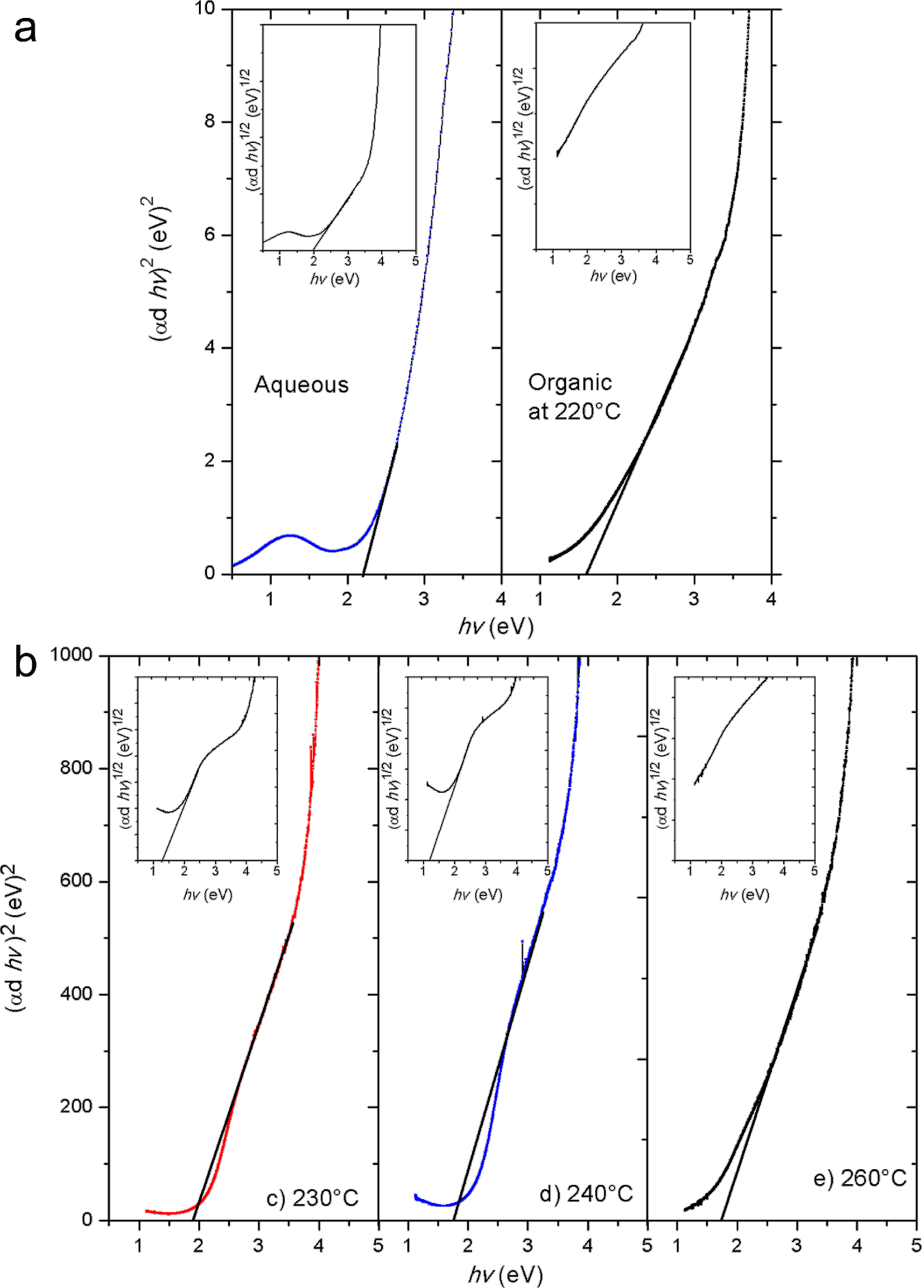
Direct band gaps of copper sulfide in a) amorphous phase obtained by aqueous synthesis and b) crystalline phases from organic media. Indirect band gap plots are included as an inset in all plots. The clear slopes in the graphics show the direct band gap energy.

The energy band gaps of the samples were computed by the Tauc plot for direct transition (Figure 5). The indirect plot (inset) did not present a satisfactory straight-line region for all samples. The Cu*_x_*S sample prepared in aqueous solution shows an *E*_g_ about 2.2 eV for the direct and 2.0 eV for the indirect transition, respectively (see inset of Figure 5). This is coherent with the value of 2.3 eV reported for crystalline or amorphous CuS covellite thin films from an aqueous solution [25,37].

On the other hand, the direct *E*_g_ values of the Cu*_x_*S samples prepared in the organic solvent are in the range of 1.57–1.87 eV. These values are adequate for an optical absorption in the visible region, which makes the samples very promising materials for solar cell applications. In Table 2 we observe a clear decrease of *E*_g_ from 1.87 to 1.60 eV from crystalline chalcocite to the digenite phase, which is in agreement to the increasing crystal size observed with TEM. These values are slightly smaller to those reported for bulk copper sulfide (1.7 and 2.0 eV) [38], so, it is consistent to the size of the nanostructures. On the other hand, an effect was found for chalcocite crystals, namely a shift into the UV region was observed and consequently, large *E*_g_ values were obtained at high deposition times without modifying the chalcocite phase [13].

### Electrical properties

**Figure 6:**
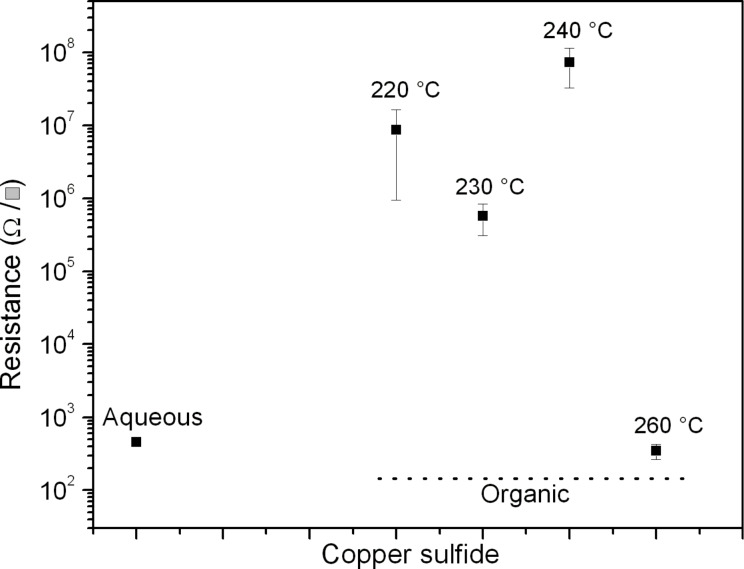
Square resistance of copper sulfide films synthesized in an aqueous solution (left) and in organic solution (right). The samples with low electrical resistance are amorphous Cu*_x_*S obtained from aqueous solution and crystalline Cu*_x_*S synthesized at 260 °C.

The Cu*_x_*S films prepared in aqueous solution are amorphous with undefined morphology. They exhibit a low square electrical resistivity (about 10^3^ Ω/sq) as shown in Figure 6. Chalcocite Cu*_x_*S from organic solution has a resistance of the order of 10^5^–10^6^ Ω/sq, while crystalline Cu*_x_*S has a resistivity of about 10^7^ Ω/sq at 240 °C and 10^2^ Ω/sq at 260 °C, respectively. In fact, the samples obtained at 230 and 240 °C, which consist of a mixture of chalcocite and digenite phases, are more resistive than the digenite phase (sample at 260 °C). This means that the copper deficiency improves the conductivity of the Cu*_x_*S, which is consistent to the reports in the literature [20]. Deficient copper structures like analite (Cu_1.75_S) have been grown onto the surface of CuS thin films, which improved their conductivity [28].

**Figure 7:**
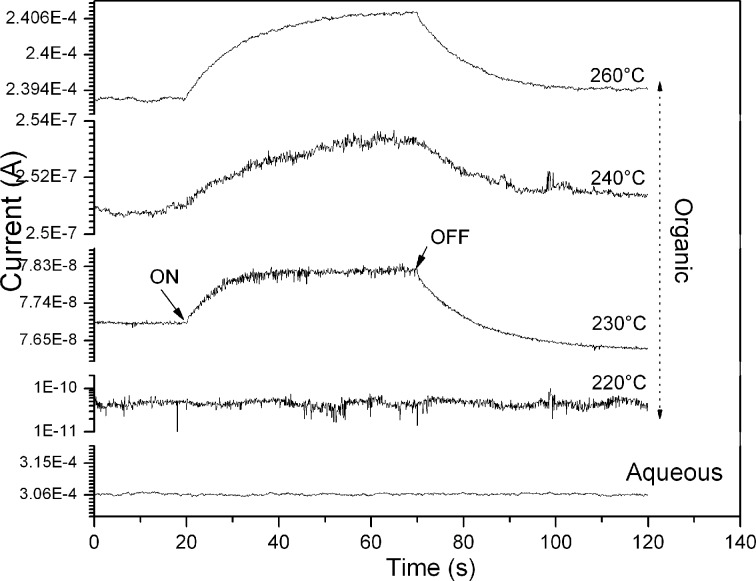
Photoconductivity of copper sulfide films, synthesized in both aqueous and organic media. Only the organic samples show photoconductivity.

The time–photo-current response of Cu*_x_*S is reported for the first time (Figure 7). It is clear that the amorphous Cu*_x_*S presents a low photosensitivity in contrast to the crystalline Cu*_x_*S samples obtained from organic solution, which are slightly photosensible, suggesting a photo-generation of carrier charges. The current increases gradually as a function of the time exposed to the light, this is attributed to the recombination of charges due to the superficial states in the Cu_x_S samples.

### Mechanism of the formation and phase transition

**Figure 8:**
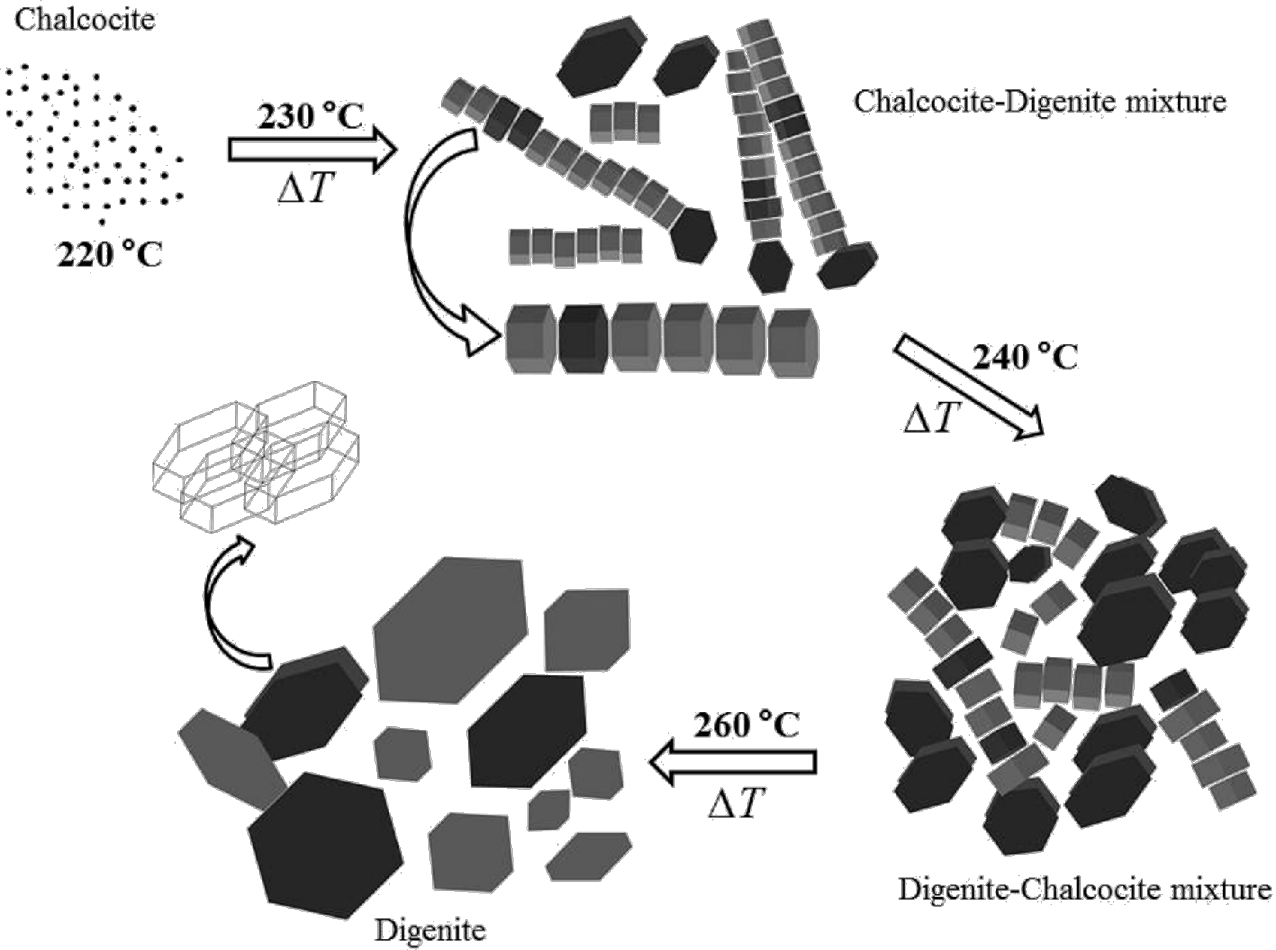
Scheme of the phase-transition mechanism from chalcocite to digenite and the formation of the respective nanocystals of the Cu*_x_*S samples as a function of the temperature. The growth of the crystalline digenite begins with the formation the nanoparticles at 220 °C and it ends at 260 °C.

According to the results presented above, a formation mechanism of the growth and the phase transition from chalcocite to digenite is proposed (Figure 8). It is clear that the nucleation of the crystals begins at 220 °C. It is a key to ensure the growth of nanoparticles at initial stages of the reaction. Above this temperature chains of aligned nanorods are formed and other crystals, nanodisks and prisms, grow. The chains of nanorods are predominant at 230 °C while nanodisks and prisms are the main morphology at 240 °C. A full phase transition from chalcocite to digenite is obtained at 260 °C.

Wang et al. obtained nanodisks of chalcocite Cu_2_S at 220 °C [15]. But, in our case, this temperature is the first stage to the phase transformation from the chalcocite to the digenite phase. According to Wang et al., the growth and rearrangement of the nanodisks are dependent on the concentration of precursors, amount of surfactant, the reaction temperature, and the reaction time. We found that this rearrangement of nanodisks is necessary for the transition of the digenite phase and it is induced only by the temperature.

On the other hand, the amorphous structure of Cu*_x_*S prepared from aqueous solution is consistent to its synthesis at low temperatures [37], during which the CuS crystalline covellite phase can be formed above 200 °C [24–25], and the tailoring of the Cu/S stoichiometric ratio and the phase transformation had been reached at temperatures between 230 to 700 °C [21]. Grozdanov and Najdoski found that the electrical sheet resistance decreases as the copper content decreased [25]. This is consistent with our results.

## Conclusion

Copper sulfide with 2 and 1.8 of Cu/S ratio were synthesized successfully from chemical synthesis in an organic solvent at 220–260 °C. Amorphous Cu*_x_*S was also obtained from aqueous solution at low temperatures with a low electrical resistance, indicative of a high conductivity. The evolution growth, formation of nanostructures, and phase transition were completely described in a scheme based on the TEM images. The full phase transition from chalcocite to digenite is obtained at 260 °C in an organic media. It is clear that the optical and electrical properties are suitable for optoelectronic applications, such as solar cells.

## Experimental

Crystalline copper sulfide nanostructures were obtained by one-pot synthesis in an organic solvent while raising the reaction temperature from 220 to 260 °C. Amorphous copper sulfide was also synthesized by a chemical reaction in aqueous solution at 40 °C. Films, colloid and powder products were obtained from both reactions.

### Reagents

For the organic reaction: copper(I) acetate (C_2_H_3_CuO_2_, Sigma-Aldrich, 97%), tri-*n*-octylphosphine oxide (OP(C_8_H_17_)_3_, TOPO Sigma-Aldrich, ≥98%), 1-dodecanethiol (C_12_H_25_SH, Aldrich, ≥98%), and dichlorobenzene (C_6_H_4_Cl_2_, Sigma-Aldrich 99%) were used as received.

The aqueous reaction: deionized water (10 MΩ·cm), thiourea (H_2_NCSNH_2_, Aldrich ≥99%), copper(II) sulfate pentahydrate (CuSO_4_·5H_2_O, Baker 99.3%), triethanolamine (TEA, C_6_H_15_NO_3_, Baker 99.8%), and sodium acetate (NaCOOCH_3_, Baker, 99.5%)

### Synthesis of nanocrystalline copper sulfide from organic solvent

It consisted of a one-pot colloidal process previously reported by Wang et al. [15] with slight modifications. In this reaction, C_2_H_3_CuO_2_ was the copper precursor and C_12_H_25_SH the sulfur precursor. In brief, 1g of TOPO and 0.0488 g of C_2_H_3_CuO_2_ were mixed with 30 mL of C_18_H_36_ in a three-neck flask. Argon was flowed into the system for 30 min to keep the reaction under an inert atmosphere. Then, the solution was heated to 160 °C and 1 mL of C_12_H_25_SH was injected quickly under vigorous stirring. The mixture reacted at constant temperature (220, 230, 240 or 260 °C) during 120 min. The colloidal brown products were washed three times with dichlorobenzene by centrifugation (20,000 rpm, 20 min) and were re-dispersed in dichlorobenzene. The organic products were cast on a Corning glass substrate and dried at 60 °C in an electric grill in order to form films.

### Synthesis of amorphous copper sulfide from aqueous solution

In this reaction thiourea and copper(II) sulfate pentahydrate (CuSO_4_·5H_2_O) were the sulfur and copper precursors, respectively, and the TEA ligand was an intermediary in the reaction. The synthesis proceeded as follows: A three-necked reactor containing 440 mL of deionized water was placed on a hot plate with magnetic stirring at 40 °C for 30 min. Clean Corning glass substrates were immersed inside the reactor in order to obtain the films by in situ deposition. Subsequently 1.3389 g of CuSO_4_·5H_2_O, previously dissolved in 20 mL of deionized water (1.3389 g/20 mL), 0.4354 g/14.5 mL of NaCOOCH_3_ and, 5.18 mL/20 mL of TEA. Finally, 0.2 g/31 mL of H_2_NCSNH_2_ was added in three aliquots each for 25 min. The substrates were withdrawn from the reactor and rinsed with deionized water. The precipitated products were washed with deionized water three times, immediately they were centrifuged and dried at room temperature. Both films and powder products, received a thermal treatment at 100 °C in air in a stove during 1 h.

### Characterization

Powders of two syntheses, aqueous and organic, respectively, of Cu*_x_*S were re-dispersed in isopropanol and toluene. One aliquot from these solutions was placed on carbon-coated copper grids for characterization by TEM, in a JEOL JEM-1010 at 80 kV of acceleration potential. Additionally, thin films of aqueous and organic syntheses of Cu*_x_*S were characterized by X-ray diffraction (Rigaku, MiniFlex, Cu Kα 1.54 Å and 2θ from 10 to 70°, rate 2°/min each 0.02 s), electrically by the four-points-probe technique, by UV–vis spectroscopy (Thermo Scientific Genesys 10S UV–vis spectrophotometer in the range of 200 to 1100 nm) in order to determine, the structural phase, the electrical resistance and optical absorbance spectra, respectively. The photoresponse measurements were made by applying a potential of 1 V at the sample: 20 s in darkness, 50 s under illumination and another 50 s in darkness. For this, two rectangular metallic contacts (0.5 × 0.2 cm) were painted on the surface of the films with silver paint in a square sample of 0.5 cm^2^.

Energy dispersive X-ray spectroscopy (EDS) was carried out in a JSM-6060LV SEM at 20 keV by using KBr pellets containing granules of Cu*_x_*S powder to make the punctual analysis.

## Supporting Information

File 1 Additional Figures.
