# Supplementary material for: Growth evolution and phase transition from chalcocite to digenite in nanocrystalline copper sulfide: Morphological, optical and electrical properties
Source: Beilstein J Nanotechnol. 2014 Sep 15;5:1542–52. doi: 10.3762/bjnano.5.166 (PMC4168957; doi:10.3762/bjnano.5.166)
Supplement: File 1 — Additional Figures. [file Beilstein_J_Nanotechnol-05-1542-s001.pdf]

# Supporting Information

for

# Growth evolution and phase transition from chalcocite to digenite in nanocrystalline copper sulfide: Morphological, optical and electrical properties

Priscilla Vasthi Quintana-Ramirez[1], Ma. Concepción Arenas-Arrocena*[2,§], José Santos-Cruz[3], Marina Vega-González[4], Omar Martínez-Alvarez[5], Víctor Manuel Castaño-Meneses[6], Laura Susana Acosta-Torres[2] and Javier de la Fuente-Hernández[2]

Address: [1]Posgrado en Ciencia e Ingeniería de Materiales, Centro de Física Aplicada y Tecnología Avanzada, Universidad Nacional Autónoma de México, 76230, Querétaro, México; [2]Escuela Nacional de Estudios Superiores Unidad León, UNAM, Boulevard UNAM No. 2011 Predio el Saucillo y el Potrero, 36969, León Guanajuato, México; [3]Facultad de Química, Materiales Universidad Autónoma de Querétaro, 76010, Querétaro, México; [4]Centro de Geociencias, UNAM, 76230, Querétaro, México; [5]Departamento de Ingeniería en Energía, Universidad Politécnica de Guanajuato, 38483, Guanajuato, México and [6]Departamento de Ingeniería Molecular de Materiales, CFATA, UNAM, 76230, Querétaro, México.

Email: Ma. Concepción Arenas Arrocena* - carenas@enes.unam.mx

*Corresponding author

§Phone: + 52 - 477 1943418

## Additional Figures

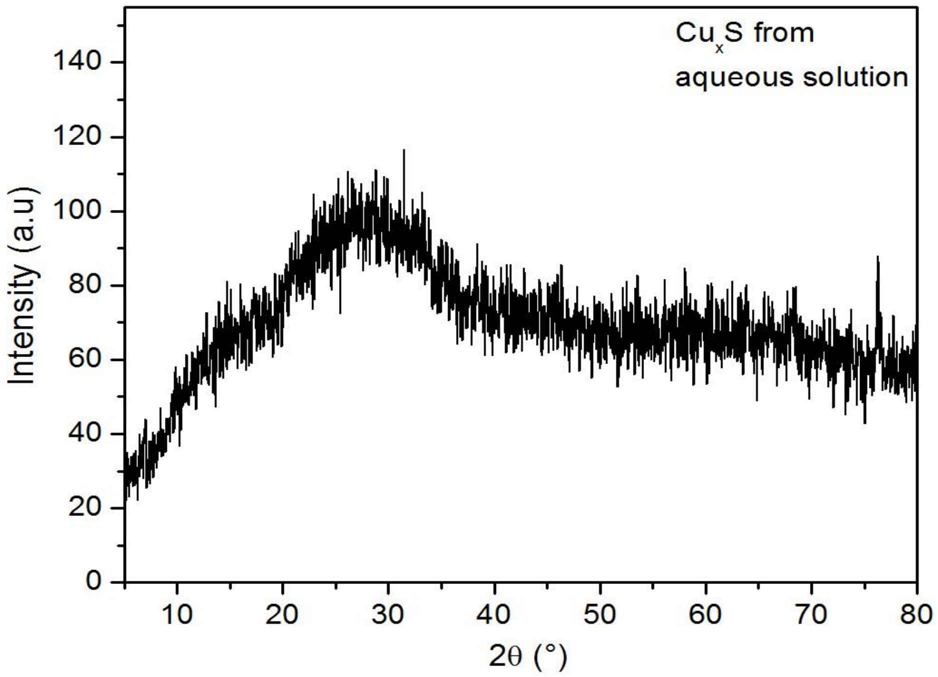


**Figure S1:** X-ray difraction of copper sulfide obtained from aqueous solution.

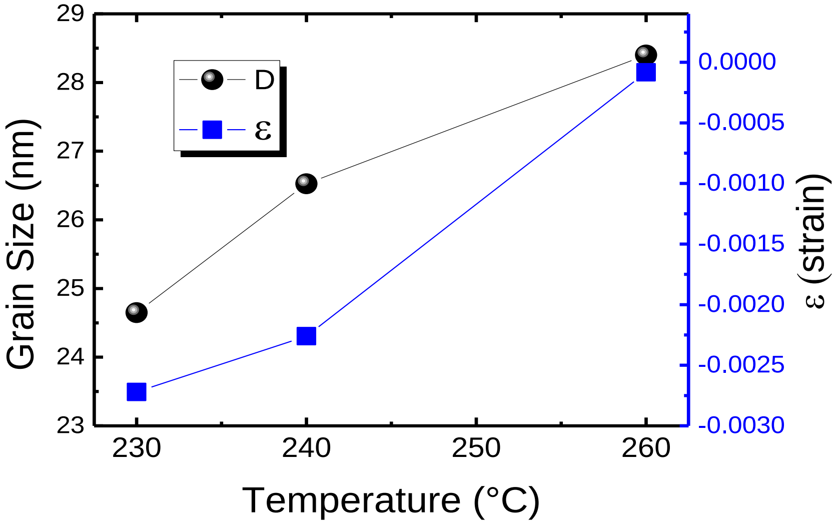


**Figure S2:** Grain size and strain the copper sulfide samples in organic solution.

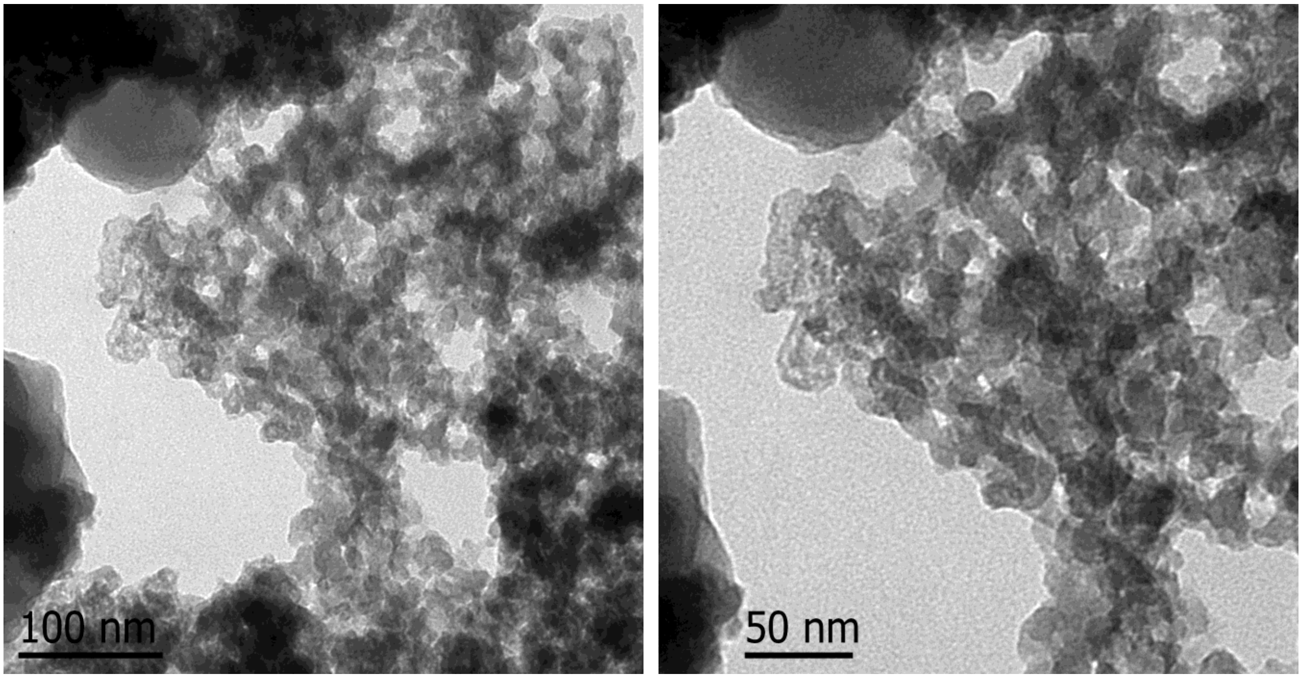


**Figure S3:** TEM images of amorphous copper sulfide prepared in aqueous solution.

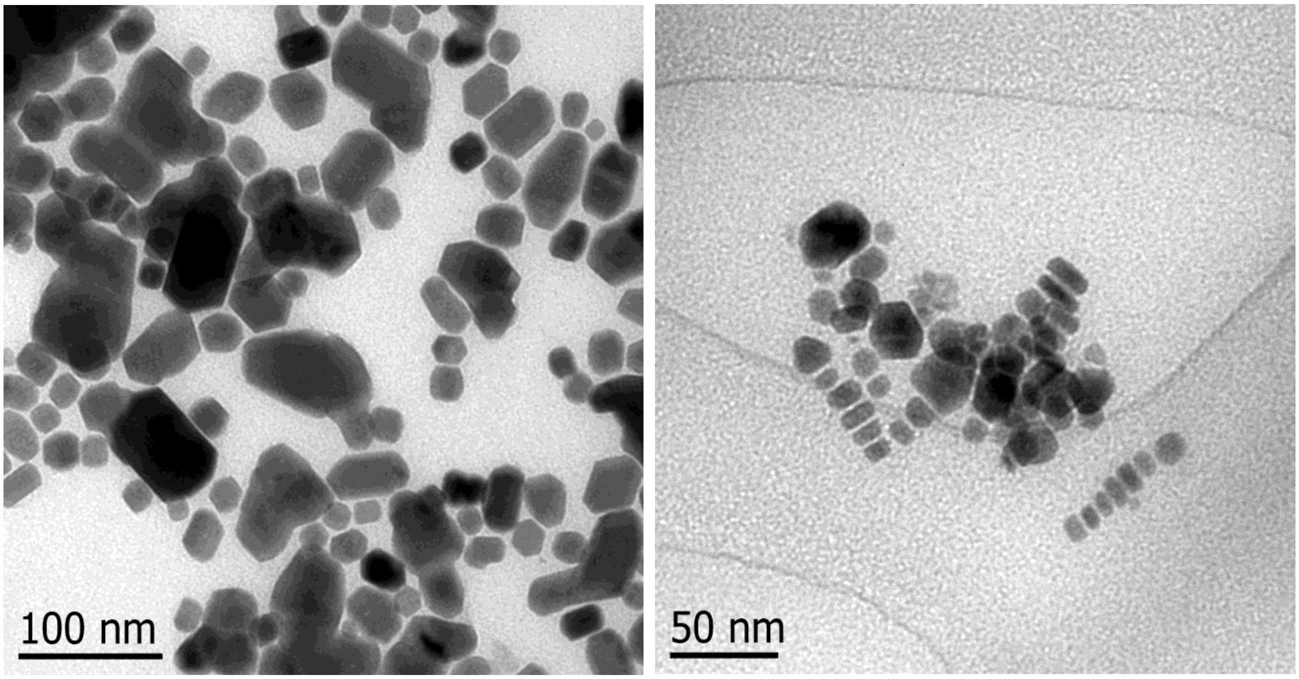


**Figure S4:** TEM images of copper sulfide prepared in organic solution at 260 °C.

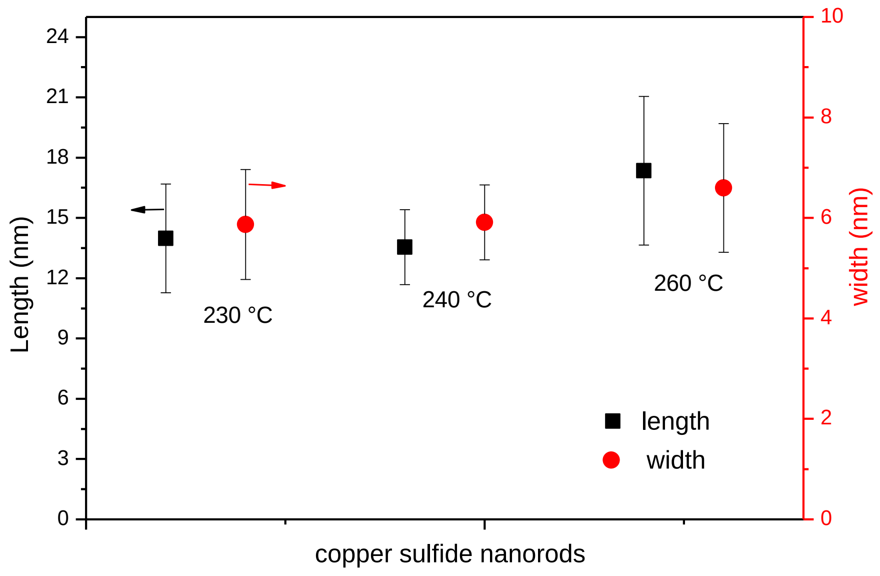


**Figure S5:** Size distribution of the $Cu_xS$ nanorods observed TEM images.
